# GPCRTree: online hierarchical classification of GPCR function

**DOI:** 10.1186/1756-0500-1-67

**Published:** 2008-08-21

**Authors:** Matthew N Davies, Andrew Secker, Mark Halling-Brown, David S Moss, Alex A Freitas, Jon Timmis, Edward Clark, Darren R Flower

**Affiliations:** 1 The Jenner Institute, University of Oxford, Compton, Newbury, Berkshire, RG20 7NN, UK; 2 Department of Computing and Centre for BioMedical Informatics, University of Kent, Canterbury, Kent, CT2 7NF, UK; 3 Department of Crystallography, Birkbeck College, University of London, Malet Street, London, WC1E 7HX, UK; 4 Departments of Computer Science and Electronics, University of York, Heslington, York, YO10 5DD, UK

## Abstract

**Background:**

G protein-coupled receptors (GPCRs) play important physiological roles transducing extracellular signals into intracellular responses. Approximately 50% of all marketed drugs target a GPCR. There remains considerable interest in effectively predicting the function of a GPCR from its primary sequence.

**Findings:**

Using techniques drawn from data mining and proteochemometrics, an alignment-free approach to GPCR classification has been devised. It uses a simple representation of a protein's physical properties. GPCRTree, a publicly-available internet server, implements an algorithm that classifies GPCRs at the class, sub-family and sub-subfamily level.

**Conclusion:**

A selective top-down classifier was developed which assigns sequences within a GPCR hierarchy. Compared to other publicly available GPCR prediction servers, GPCRTree is considerably more accurate at every level of classification. The server has been available online since March 2008 at URL: .

## Background

The G protein-coupled receptors (GPCR) comprise a diverse range of integral membrane proteins regulating many important physiological functions [1-3]. Ligand binding to a GPCR on the cell surface initiates cell signaling. An extremely heterogeneous set of molecules act as GPCR ligands. The GPCRs are a common target for therapeutic drugs and approximately 50% of all marketed drugs target GPCRs [4,5]. In spite of their functional and sequence diversity, GPCRs share certain common structural features, but show a far greater conservation of three-dimensional structure than primary sequence [6]. This makes it difficult to develop for GPCR subtypes a comprehensive classification system based on sequence [7]. The most commonly-used system of classification is that implemented in the GPCRDB database [8], which divides the GPCRs into six classes (Class A: Rhodopsin-like, with over 80% of all GPCRs in humans; Class B: Secretin-like; Class C: Metabotropic glutamate receptors; Class D: Pheromone receptors; Class E: cAMP receptors; and the much smaller Class F: Frizzled/smoothened family). Classes A, B, C and F are found in mammalian species while Class D proteins are found only in fungi and Class E proteins are exclusive to *Dictyostelium*. The six classes are further divided into sub-divisions and sub-sub-divisions based on the function of a GPCR and its specific ligand.

Previous attempts at classifying the GPCRs from its primary sequence have included motif-based classification tools [9,10] and machine learning methods such as Hidden Markov Models [11,12] and Support Vector Machines (SVMs) [13]. Several publicly-available SVM-based GPCR classifiers exist: PRED-GPCR [14,15], GPCR-PRED [16] and GPCRsclass [17]. Some predictive techniques have used a combination of SVMs and HMMs [18]. Other approaches towards GPCR Classification have included Self-Organising Maps [19], Quasi-predictor Feature Classifiers [20] and Decision Trees [21]. GPCRTree is a new publicly-available server based on the idea of selecting the best classifier (from a set of candidate classifiers) at each node of the GPCR class tree.

## Findings

### Algorithm

A previously-constructed comprehensive GPCR sequences dataset was used to train and test the classifier [22]. Proteins shorter than 280 amino acids were removed, eliminating incomplete protein sequences. All identical sequences were removed to avoid redundancy and classes with fewer than 10 examples were also removed. The dataset used to train the server contains 8222 protein sequences in 5 classes at the family level (A-E), 38 classes at the sub-family level, and 87 classes at the sub-subfamily level. Class F was not considered since it contains too few sequences to develop an accurate classification model. The system uses an alignment-independent classification system based on amino acids physical properties. Proteochemometrics uses 5 "z-values" (z1–z5) derived from 26 real physiochemical properties using principal component analysis [23,24]. These five values are calculated for each amino acid in the sequence and are used to generate the 15 attribute values described in [17], giving a purely numerical description of the protein.

The GPCRTree server classifies at the GPCR Class, Subfamily and Sub-Subfamily level. Hierarchical classification of a sequence is performed using a selective top-down approach, whereby each group of sibling nodes in the GPCR class tree becomes a flat classification problem solved using a standard classifier [25,26], obviating the need to devise a novel classifier. The full dataset trains the root classifier, while only relevant subsets of the data are used to train classifiers at the subfamily and sub-subfamily levels. When an unclassified sequence is presented to the algorithm, the root level classifier assigns it to a class, which is then passed down to an appropriate classifier at the next level until it is assigned to a subfamily and a sub-subfamily [27]. Instead of a single classification algorithm being used at each node of the class tree, many classifiers are trained using a subset of the training set called the sub-training set, and then tested using a separate part of the training set called the validation set. The classifier with the highest classification accuracy on the validation set is selected for that node. Eight standard classification algorithms were used as candidate classifiers at each node of the GPCR tree. All code was written using the open source WEKA data mining package [28,29] and the default parameters were used for each algorithm.

### Testing

The GPCRTree server has been validated against three other predictive GPCR servers [22]. The GPCRTree server was trained using the full GPCRtree dataset, and then tested with each GPCR server dataset as test data. GPCRTree produced accuracies of 97% at the Class level, 84% at the Sub-family and 75% at the Sub-Subfamily level. This exceeded the PRED-GPCR server at the Class level and is comparable at the Sub-family level. It exceeds the GPCRPred server at all levels of the hierarchy. The GPCRsclass server was the most successful classifier at the most specific (sub-sub-family) level; this may be because the classifier is overly specialised, being applicable only to the Class A Amine sub-subfamily level. Of servers applicable to all GPCR classes, GPCRTree is the most accurate GPCR prediction server currently available.

### Implementation

GPCRTree is available through a web interface – . It was implemented using PHP, dHTML and a java client. The PHP interface affords a simple and straightforward method to submit a protein sequence for evaluation. The code for the selective top-down approach, as previously published, required several changes to facilitate its effective integration into the server environment. Training was modified such that all GPCR proteins belonging to a class with 10 or more examples (protein sequences) were used. The algorithm then pauses and waits for input that will come as an auxiliary program making a TCP socket connection with the selective top down classifier. Upon connection, the auxiliary program will send the protein sequence to be classified and then pause. The classifier will make a prediction and then return the result. A TCP connection has been used for several reasons. It can allow multiple users to access the classifier. Separate users can run separate auxiliary programs, and so the classifier can queue these requests ensuring that only one will invoke the classifier at any given time. The remainder will be queued and serviced in the order of submission. Moreover, this architecture promotes portability. It may be necessary, for resource or security reasons, to run the classifier on different hardware. In this case, the server can invoke the auxiliary program which can connect via network connection to the separate machine running the classifier.

**Figure 1:**
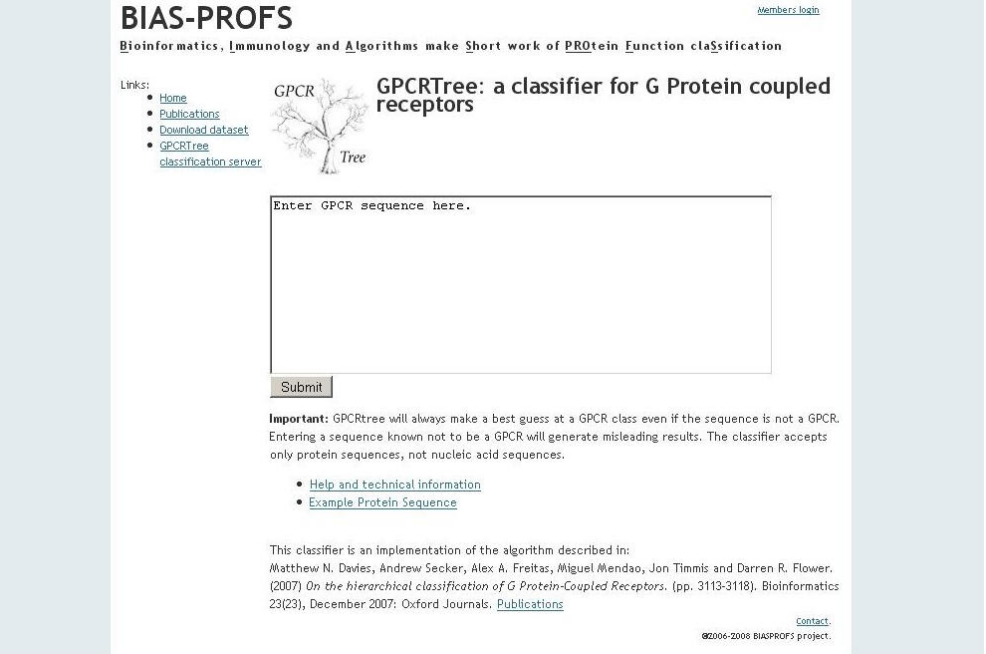
Input page for GPCRTree server.

**Figure 2:**
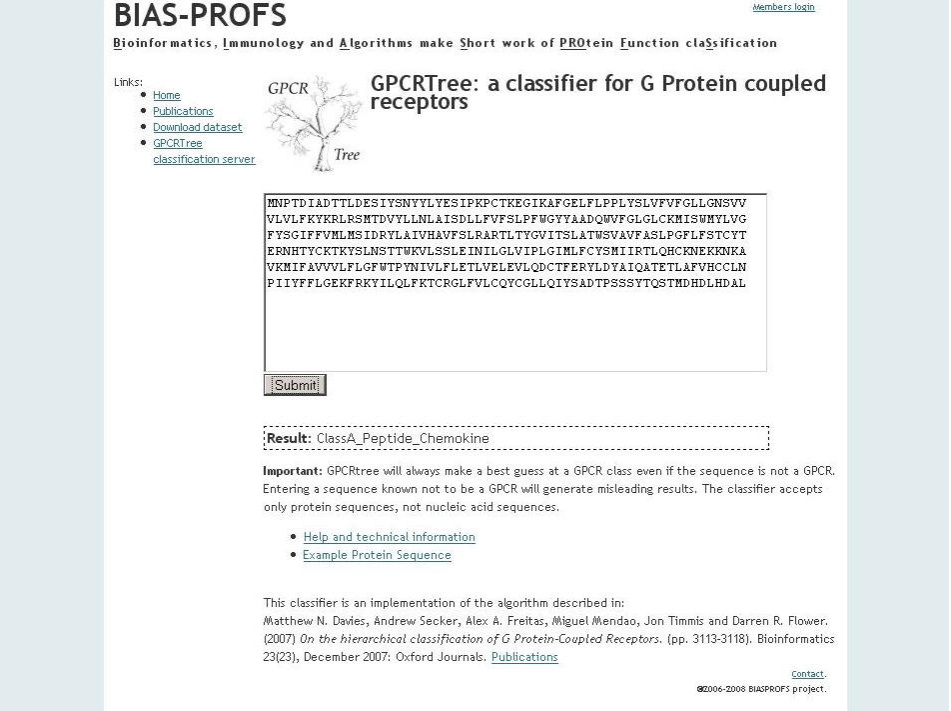
Results page for the GPCRTree server showing the prediction for the sequence of Chemokine CCR4 receptor.

A user enters a protein sequence in plain or fasta format and submits the job (Figure 1). The interface then sends an AJAX call to the java client. The GPCRTree java client submits sequences to the GPCRTree server, where they are classified and the classification returned to the java client which in turn passes this result to the interface. The sequence and classification is then displayed below the submission button (Figure 2).

Where non-standard residues are included within the sequences, substitutions are made: a sequence containing a 'B' (asparagine or aspartic acid) is assigned as an asparagine 'N'; a 'Z' (glutamine or glutamic acid) is assigned as a glutamine 'Q'; and a 'U' (selenocysteine) is assigned as a cysteine 'C'. All unknown residues 'X' were assigned as alanines 'A'.

## Conclusion

GPCR classification is among the most challenging problems in bioinformatics due to the sequence diversity of the GPCR superfamily and the uneven distribution of its various family subgroups. GPCRTree is the first server to implement an alignment-independent representation of protein sequences and is also the first to classify sequences using a classifier specifically selected for each group of sibling nodes in the GPCR functional classification tree. By selecting the best classifier (from a set of candidate classifiers) at each GPCR class tree node, the selective top-down method effectively exploits the fact that different classifiers have different biases that are more suitable for different classification problems. GPCRTree is currently the most accurate publicly-available server for the prediction of GPCR sequence classification and it utilises a simple yet robust interface that can undertake multiple classifications simultaneously.

## Availability and requirements

**Project name: **GPCRTree

**Project home page: **

**Operating system(s): **Platform independent

**Programming language: **PHP, dHTML, Java

**Other requirements: **None

**License: **None

**Any restrictions to use by non-academics: **None

## Abbreviations

GPCR: G protein coupled receptor; TCP: Tranmission Control Protocol; WEKA: Waikato Environment for Knowledge Analysis; SVM: Support Vector Machine

## Competing interests

The authors declare that they have no competing interests.

## Authors' contributions

MD Built GPCRtree datatset, created alignment-free representations of protein sequences, and wrote the paper. AS Designed and implemented the selective top-down method for hierarchical classification. Implemented method of turning raw protein sequences into numerical attributes. Assisted in writing the paper and implementation of the code on the GPCRTree web server. MHB Constructed and implemented GPCRTree server, currently maintains server at Birkbeck College, University of London DM Supervised construction of GPCRTree server at Birkbeck College AF Supervised the design of the selective top-down method for hierarchical classification. JT Supervised a mathematical analysis of data mining algorithms for hierarchical classification EC Performed a mathematical analysis of data mining algorithms for hierarchical classification. DRF Supervised design and construction of GPCRtree dataset, development of representations of protein sequences, and co-wrote the paper. All authors read and approved the paper.
